# African swine fever virus A151R protein antagonizes the antiviral activity of barrier-to-autointegration factor (BAF) by targeting its dsDNA-binding activity

**DOI:** 10.1128/jvi.00988-26

**Published:** 2026-09-11

**Authors:** Kepalee Saeng-chuto, Zhigang Wang, Kaylee Bargeron, Matthew S. Wiebe

**Affiliations:** 1 School of Veterinary Medicine and Biomedical Sciences, University of Nebraska 14719, Lincoln, Nebraska, USA; 2 Nebraska Center for Virology, University of Nebraska 14719, Lincoln, Nebraska, USA; 3 School of Biological Sciences, University of Nebraska 118735, Lincoln, Nebraska, USA; Northwestern University Feinberg School of Medicine, Chicago, Illinois, USA

**Keywords:** BAF, A151R, vaccinia, poxviruses, African swine fever virus, antiviral activity, dsDNA viruses

## Abstract

Barrier-to-autointegration factor (BAF) is a ubiquitous double-stranded DNA-binding protein that compacts DNA and can restrict poxvirus replication in the cytoplasm. BAF antiviral DNA-binding activity is tightly regulated by dynamic phosphorylation mediated by viral and cellular enzymes. For example, vaccinia virus counteracts BAF by encoding the B1 kinase, which phosphorylates BAF and abrogates its DNA-binding activity. Some DNA viruses, such as African swine fever virus (ASFV), undergo cytoplasmic replication but appear to lack a B1-like kinase. Interestingly, ASFV encodes A151R, a viral protein recently found to stably interact with BAF. Here, we demonstrate that A151R is capable of counteracting the antiviral properties of BAF. Structural modeling indicates that A151R is not a protein kinase and does not phosphorylate BAF but instead directly targets its double-stranded DNA-binding interface. This interaction enhances genome replication and progeny production of a B1-deficient virus. Mechanistically, A151R markedly impairs BAF DNA binding and disrupts its dimerization, a key requirement for high-affinity DNA association. Importantly, disruption of the A151R-BAF interaction abolishes these effects and restores BAF antiviral function. In addition, expression of the unphosphorylatable BAF mutant, which normally exhibits strong chromatin association, was redistributed to the cytoplasm in the presence of A151R, further supporting phosphorylation-independent regulation of BAF-DNA association. In conclusion, our findings support a previously unrecognized mechanism by which ASFV A151R disables BAF antiviral activity by obscuring its DNA-binding interface and inhibiting DNA binding in a phosphorylation-independent manner.

**IMPORTANCE:**

DNA viruses replicating in the cytoplasm must overcome host intrinsic defenses to ensure productive replication, yet the mechanisms underlying their antagonism of the DNA-binding antiviral factor BAF remain incompletely understood. Here, we identify African swine fever virus (ASFV) A151R as a novel viral regulator that disables BAF by targeting its double-stranded DNA-binding interface rather than altering its phosphorylation state. We demonstrate that A151R impairs BAF DNA binding, disrupts its dimerization, and promotes viral DNA accumulation and progeny production in a BAF-dependent manner. Importantly, this activity requires A151R-BAF interaction and is independent of BAF phosphorylation status. Our findings reveal a previously unrecognized strategy employed by ASFV to neutralize host DNA-binding restriction factors and expand the molecular framework of BAF-mediated antiviral defense.

## INTRODUCTION

BAF is a small (10 kDa), highly conserved metazoan protein encoded by the BANF1 gene that consists of 89 amino acids and localizes to both the nucleus and cytoplasm (1). Nuclear BAF maintains genome integrity in uninfected cells via key roles during mitosis and other processes, whereas cytoplasmic BAF recognizes and compacts foreign double-stranded DNA (dsDNA) in a sequence-independent manner (2–7). A previous study showed that cytoplasmic BAF rapidly relocalizes to transfected DNA from diverse sources, including plasmid DNA, *Escherichia coli* DNA, and vaccinia virus DNA, demonstrating its ability to recognize foreign DNA from multiple sources (8). Through recognition and compaction of foreign DNA, cytoplasmic BAF serves as a formidable antiviral defense against poxviruses and likely cytoplasmic DNA viruses in general.

BAF dsDNA-binding activity is regulated through reversible phosphorylation (9–12). In particular, BAF is phosphorylated at its N terminus by cellular kinases called vaccinia-related kinases (VRKs), which leads to its dsDNA dissociation (9). Furthermore, previous studies demonstrated that hyperphosphorylated BAF exhibits reduced DNA-binding activity, impaired dimerization, and increased cytoplasmic localization, correlating with a loss of antiviral activity (13). Conversely, BAF is dephosphorylated by serine/threonine protein phosphatase 2A (PP2A) in cooperation with ankyrin repeat and LEM domain-containing protein 2 (ANKLE2), thereby restoring its dsDNA-binding and antiviral activity (7, 10–12). Together, these observations indicate that BAF antiviral activity depends on coordinated dynamic regulation of its phosphorylation state, DNA-binding domain, dimerization interface, and subcellular localization.

Poxviruses must counteract BAF-mediated antiviral restriction to ensure productive infection. Vaccinia virus, a prototype poxvirus, is a large dsDNA virus with a ~195-kb genome encoding approximately 200 proteins and completing its entire life cycle in the cytoplasm of infected cells (14). During infection, cytoplasmic BAF recognizes and binds viral DNA, thereby restricting viral DNA replication (2–6, 15–17), as supported by the observation that BAF depletion rescues vaccinia virus replication (5–7). To counteract this antiviral defense, vaccinia virus encodes the B1 protein, a serine/threonine kinase that functionally mimics host VRKs to phosphorylate and inactivate BAF, thereby suppressing its antiviral activity and promoting viral replication (5, 6, 8, 18–20). Previous studies have shown that B1 kinase activity is essential for vaccinia virus replication, as viral replication is dramatically reduced upon mutation (Cts2) or deletion (ΔB1) of B1 (5, 6, 8, 16, 18–21) and can be restored by overexpression of host VRKs (16, 21). In contrast, ANKLE2-mediated dephosphorylation of BAF is also associated with reduced ΔB1 vaccinia virus replication (7). Together, these findings suggest that ΔB1 vaccinia virus replication correlates inversely with BAF activity, as viral DNA replication is highly sensitive to active BAF-mediated restriction (6). These properties make the ΔB1 virus a useful model for investigating how BAF’s antiviral activity may be targeted by other putative regulators of either cellular or viral origin.

African swine fever virus (ASFV), the causative agent of African swine fever in swine, is a large, enveloped dsDNA virus and the sole member of the family *Asfarviridae* (22). Its genome is approximately 170–190 kb and encodes around 150–170 proteins, including more than 68 structural proteins and over 100 non-structural proteins involved in viral assembly, genome replication and repair, immune evasion, and host cell manipulation (22–24). Similar to vaccinia virus, ASFV also replicates its genome in the cytoplasm of infected cells (25), raising the possibility that cytoplasmic BAF may contribute to ASFV restriction. Notably, unlike vaccinia virus, ASFV does not appear to encode a B1-like kinase to inactivate BAF, prompting us to investigate how ASFV evades BAF binding to viral DNA. Among the candidate ASFV genes that have been functionally studied, A151R is a non-structural protein implicated in ASFV replication and virulence, as its depletion reduces viral replication (26–28). Recently, A151R has been reported to directly bind BAF (28), raising the possibility that ASFV employs A151R to counteract BAF-mediated antiviral restriction. Here, we demonstrated that ASFV A151R targets the BAF DNA-binding interface, impairing BAF dimerization, DNA-binding ability, and antiviral function in the context of a B1-deficient vaccinia infection. These impacts of A151R occur independently of the phosphorylation status of BAF, revealing that this ASFV protein utilizes a novel mechanism in its inhibition of BAF’s intrinsic immune activity.

## RESULTS

### A151R modeling predicts interaction with BAF at its dsDNA-binding surface

**Fig 1:**
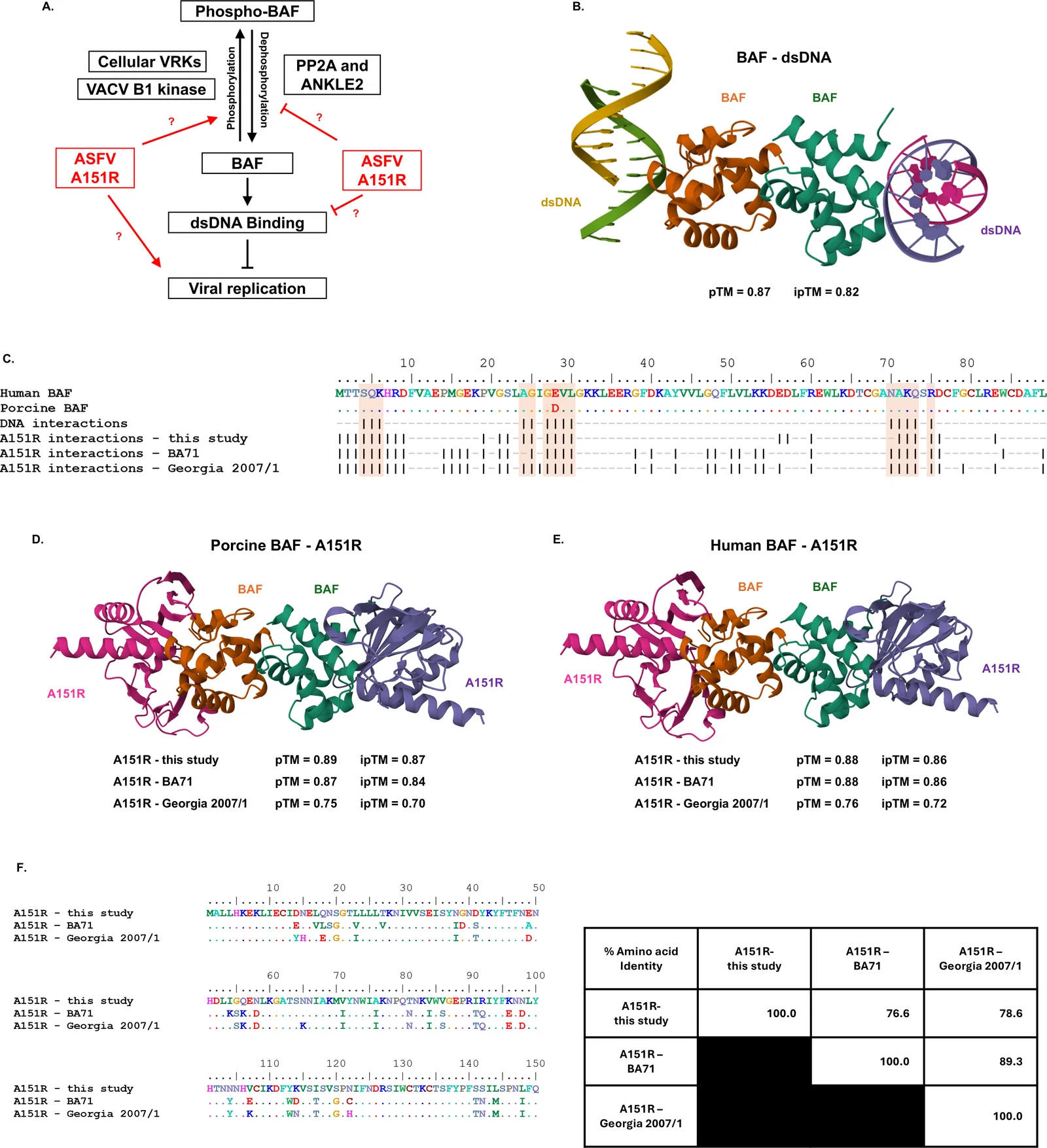
Model and structural prediction of A151R–BAF interaction. (**A**) BAF inhibits vaccinia virus DNA replication through its dsDNA-binding activity. BAF function is regulated by phosphorylation and dephosphorylation. PP2A-ANKLE2-mediated dephosphorylation promotes BAF binding to dsDNA, whereas phosphorylation by cellular VRKs or the vaccinia virus kinase B1 reduces its DNA-binding activity and prevents viral DNA compaction. Unlike vaccinia virus, ASFV lacks a B1-like kinase, raising the question of how ASFV counteracts BAF-mediated restriction. (**B**) Predicted BAF-DNA interaction model generated using AlphaFold 3. (**C**) Amino acid sequence alignment of porcine and human BAF performed using BioEdit. Predicted BAF-DNA and BAF-A151R interaction residues identified using FoldScript are indicated by vertical bars (|). A151R variants used for prediction include the A151R used in this study, BA71, and Georgia 2007/1 strains. Residues within the BAF dsDNA-binding region predicted to interact with A151R are highlighted in light orange. (**D and E**) Predicted interactions between A151R and porcine (**D**) or human (**E**) BAF generated using AlphaFold 3. The predicted template modeling (pTM) score reflects overall model accuracy, whereas the interface predicted template modeling (ipTM) score indicates confidence of protein-protein interface prediction. Scores range from 0 to 1, with higher values indicating greater confidence. (**F**) Alignment (left) and percentage amino acid identity (right) of the A151R amino acid sequence used in this study compared with representative BA71 and Georgia 2007/1 A151R variants.

Vaccinia virus encodes the B1 kinase, which mimics host VRKs to phosphorylate BAF, thereby suppressing its dsDNA-binding activity and facilitating viral replication (5, 6, 8, 18–20) (Fig. 1A). In contrast, ASFV lacks a B1-like kinase to inactivate BAF. Considering the previous report using both yeast two-hybrid analysis and microscale thermophoresis analysis supporting a direct interaction between A151R and BAF (28), we hypothesized that A151R may modulate BAF-dependent DNA binding and thereby influence viral replication (Fig. 1A). To test this, we established a comparative system using human cell lines and a ΔB1 vaccinia virus mutant. Human cells were selected based on their prior use with ΔB1, and comparison of porcine BAF (UniProtKB: F1RU33) and human BAF (UniProtKB: O75531) revealed only a single amino acid difference at position 28 and 98.8% sequence identity (Fig. 1C), indicating strong conservation. We then performed structural modeling of BAF in complex with dsDNA or A151R (UniProtKB: P0CA55) using AlphaFold 3 (29). We further assessed whether the AlphaFold-modeled A151R and BAF structures were consistent with published crystal structures of each of these proteins alone. PyMOL structural alignment of the AlphaFold-derived A151R with the crystal structure of A151R (PDB: 7CMA) revealed a root-mean-square deviation (RMSD) of 0.787 Å over 137 atoms, and an alignment of the AlphaFold-derived BAF dimer with the crystal structure of human BAF (PDB: 1CI4) revealed an RMSD of 0.251 Å over 153 atoms. The low RMSD (0–1 Å) indicates that the A151R-BAF model produced by AlphaFold 3 exhibits strong consistency with prior crystallization data (30).

Interface mapping using FoldScript (31) found that human BAF is predicted by AlphaFold to engage dsDNA through several residues, including Ser4, Gln5, Lys6, Ala24, Gly25, Gly27, Asp28, Val29, Leu30, Asn70, Ala71, Lys72, Gln73, and Arg75 (Fig. 1B and C), consistent with previous structural data (2). The AlphaFold results also yielded a high-confidence prediction that A151R binds porcine BAF (predicted template modeling [pTM] = 0.89 and interface predicted template modeling [ipTM] = 0.87) (Fig. 1D) or human BAF (pTM = 0.88 and ipTM = 0.86) (Fig. 1E) at the completely conserved dsDNA-binding region of BAF (pTM = 0.87 and ipTM = 0.82) (Fig. 1B and C). Finally, we also investigated whether these residues exhibit sequence differences among A151R variants. Specifically, the A151R proteins expressed by the BA71 (GenBank: AKO62719.1) and Georgia 2007/1 (GenBank: CAD2068398.1) strains were included as comparison sequences. The A151R proteins encoded by the BA71 and Georgia 2007/1 strains shared 76.6% and 78.6% amino acid sequence identity, respectively, with the A151R sequence used in this study (Fig. 1F). AlphaFold 3 predicted that A151R in both strains retains the predicted interaction with the dsDNA-binding region of BAF (Fig. 1C). For BA71, the confidence scores were pTM = 0.87 and ipTM = 0.84 with porcine BAF (Fig. 1D) and pTM = 0.88 and ipTM = 0.86 with human BAF (Fig. 1E). For Georgia 2007/1, the corresponding scores were pTM = 0.75 and ipTM = 0.70 with porcine BAF (Fig. 1D), and pTM = 0.76 and ipTM = 0.72 with human BAF (Fig. 1E). Together, these structural models are strongly suggestive that A151R retains its interaction with BAF regardless of viral strain or BAF species and may interfere with BAF’s association with DNA.

### Expression of ASFV A151R protein promotes ΔB1 vaccinia virus replication

**Fig 2:**
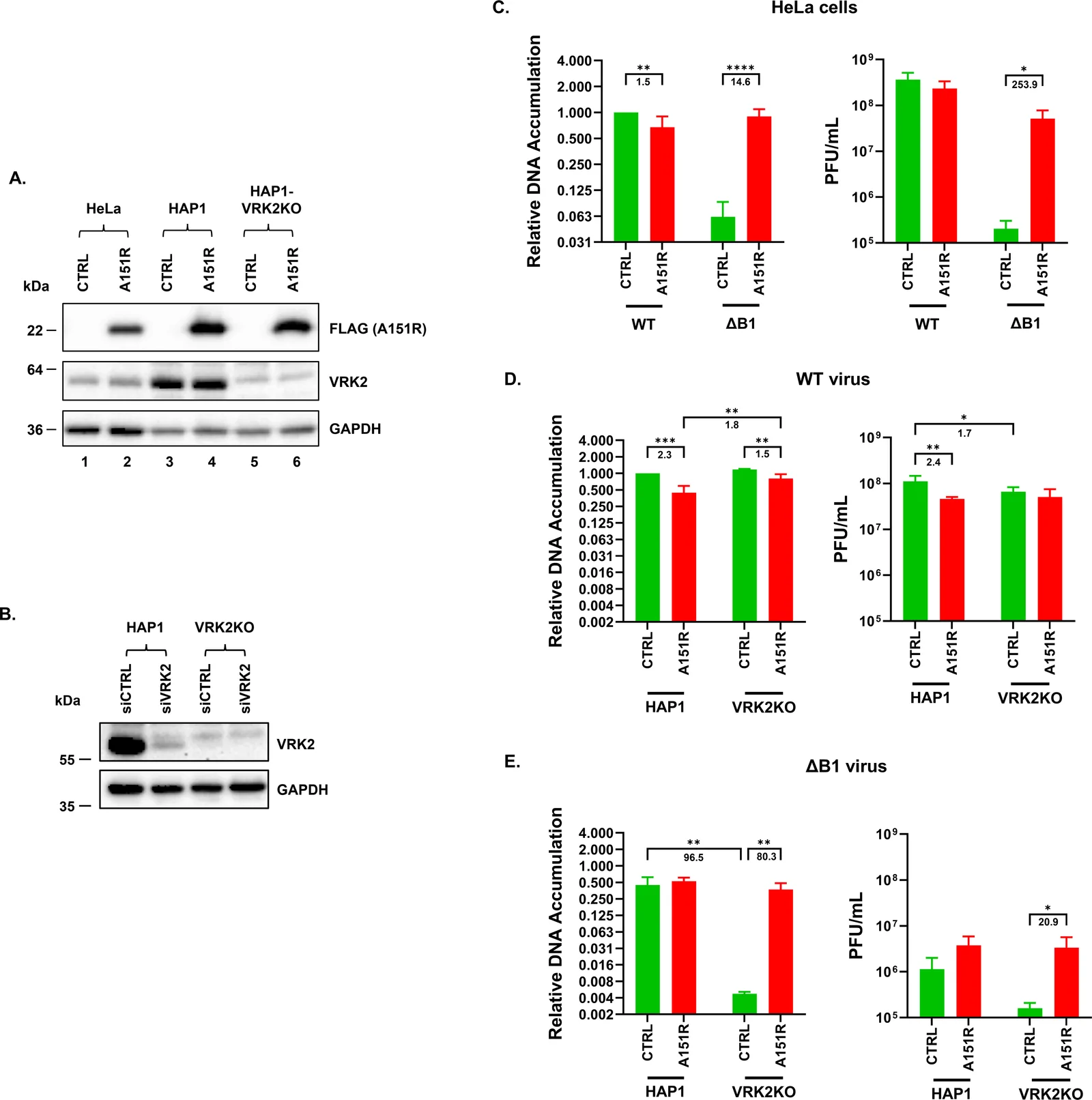
Impact of ASFV A151R expression on WT and ΔB1 vaccinia virus replication in HeLa and HAP1 cells. (**A**) HeLa, HAP1, and HAP1-VRK2KO cells were individually transduced with lentivirus expressing A151R. Control cell lines transduced with lentivirus carrying the empty vector (CTRL) were also generated. Cells were then treated with 0.5 µg/mL doxycycline for 6 h to induce protein expression and analyzed by immunoblotting. GAPDH served as a loading control. (**B**) HAP1 and HAP1-VRK2KO cells were transfected with siCTRL or siVRK2 and analyzed by immunoblotting. (**C**) DNA accumulation and viral yield were measured for WT or ΔB1 vaccinia virus at 24 hpi in transduced HeLa cells infected at an MOI of 3. (**D and E**) DNA accumulation and viral yield were measured for WT (**D**) or ΔB1 (**E**) vaccinia virus at 24 hpi in transduced HAP1 and HAP1-VRK2KO cells infected at an MOI of 3. Data are from more than three independent experimental replicates. *, *P*  <  0.05; **, *P*  <  0.01; ***, *P*  <  0.001; ****, *P*  <  0.0001. Relative fold differences are given under each bracket.

We next investigated whether A151R affects replication of wild-type (WT) and ΔB1 vaccinia virus, a BAF-restricted virus. To this end, we generated HeLa and HAP1 cell lines stably expressing N-terminal FLAG-tagged A151R using a doxycycline-inducible lentiviral system. Cells transduced with an empty vector (CTRL) were used as controls. Because endogenous VRK2 can compensate for loss of B1 kinase activity during vaccinia virus infection in HAP1 cells, particularly during viral DNA replication (21), VRK2 knockout HAP1 cells (HAP1-VRK2KO) were also included. Stable expression of A151R was confirmed by immunoblotting (Fig. 2A). A non-specific band detected in the VRK2 immunoblot of HAP1-VRK2-KO cells (Fig. 2A, lanes 5–6) was confirmed to be unrelated to VRK2, as its level was not reduced following transfection with a previously validated small interfering RNA targeting VRK2 (siVRK2) (32) and remained comparable to that in cells treated with control siRNA (siCTRL) (Fig. 2B). Following 6 h of doxycycline induction, cells were infected with either WT or ΔB1 vaccinia virus at a multiplicity of infection (MOI) of 3 PFU per cell. At 24 h post-infection, A151R expression had minimal effects on WT vaccinia virus DNA accumulation or progeny production (Fig. 2C and D). In contrast, A151R expression markedly increased ΔB1 vaccinia virus DNA accumulation in HeLa (14.6-fold) (Fig. 2C, left) and HAP1-VRK2KO cells (80.3-fold) (Fig. 2E, left). This effect was also reflected in viral progeny production, with ΔB1 virus titers increased in HeLa (253.9-fold) (Fig. 2C, right) and HAP1-VRK2KO cells (20.9-fold) (Fig. 2E, right) upon A151R expression. Notably, A151R expression restored ΔB1 vaccinia virus replication in HAP1-VRK2KO cells to levels comparable to those observed in parental HAP1 cells (Fig. 2E), indicating that the effect of A151R is independent of VRK2-mediated compensation and suggests targeting of BAF-dependent restriction. The selective enhancement of ΔB1 virus replication, but not WT virus, further suggests that A151R counteracts the BAF-mediated antiviral restriction normally overcome by the vaccinia virus B1 kinase.

### A151R binding to BAF is required for rescue of BAF-sensitive virus replication

**Fig 3:**
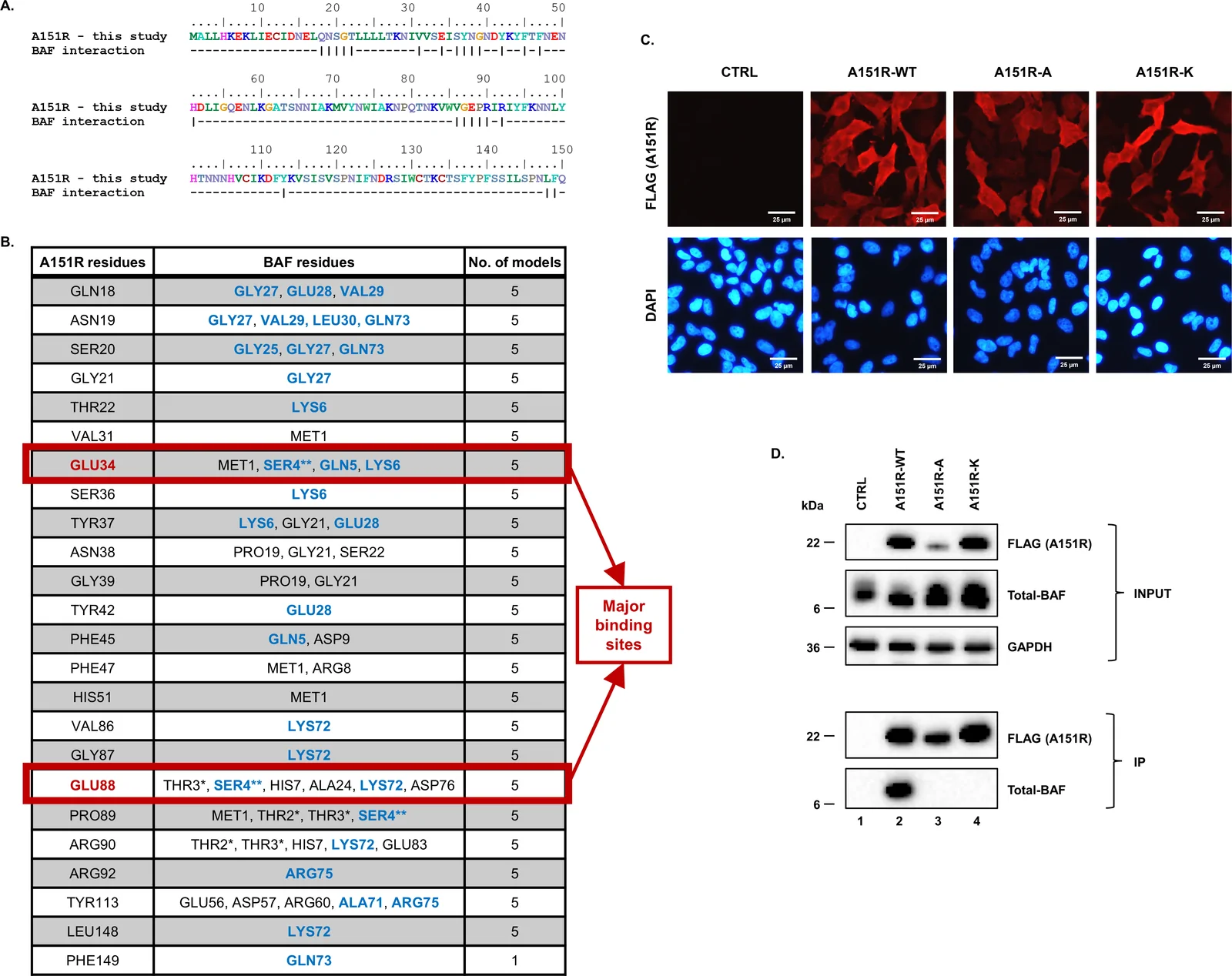
Identification of the A151R-BAF binding interface. (**A and B**) Predicted protein-protein interaction interface between A151R and BAF. (**A**) Predicted A151R-BAF interaction residues identified using FoldScript are indicated (|). (**B**) Red residues indicate glutamic acid 34 (Glu34) and glutamic acid 88 (Glu88), which were predicted as major A151R binding hotspots for interaction with BAF. Blue residues indicate the predicted dsDNA-binding interface of BAF. * indicates threonine 2 (Thr2) and threonine 3 (Thr3), and ** indicates serine 4 (Ser4), the primary phosphorylation site of BAF. (**C**) HeLa cells were transduced with lentiviral constructs expressing either FLAG-tagged wild-type A151R (A151R-WT), an alanine-substitution mutant in which Glu34 and Glu88 were replaced with alanine (A151R-A), or a lysine-substitution mutant in which Glu34 and Glu88 were replaced with lysine (A151R-K). Control cell lines transduced with lentivirus carrying the empty vector (CTRL) were also generated. Cells were analyzed by immunofluorescence using anti-FLAG antibody (red), with nuclei counterstained with DAPI (blue). Images were acquired using the automatic exposure setting of the imaging system. (**D**) Coimmunoprecipitation analysis of A151R-BAF interaction. Cell lysates from doxycycline-induced HeLa cells expressing FLAG-tagged A151R constructs were immunoprecipitated using anti-FLAG antibody-conjugated beads and analyzed by immunoblotting with antibodies against FLAG and BAF. INPUT samples were included to confirm protein expression levels. GAPDH served as a loading control. Data are from more than three independent experimental replicates. A representative immunoblot is shown with lane numbers shown below each column.

We next asked whether the A151R-BAF interaction is required for promoting viral replication. To address this, we analyzed the predicted protein-protein interface using FoldScript, which identified candidate A151R-BAF contact sites, with residues E34 and E88 of A151R representing major binding hotspots (Fig. 3A and B). These residues were predicted to engage BAF phosphorylation sites, including Ser4, the primary site, and Thr3 (9), as well as its dsDNA-binding region (Fig. 3A and B). To validate these predictions, we generated HeLa cell lines stably expressing N-terminal FLAG-tagged A151R wild-type (A151R-WT) or mutant constructs in which glutamic acid residues at positions 34 and 88 (Glu34 and Glu88) were substituted with either alanine (A) or lysine (K) (A151R-A and A151R-K), using an identical doxycycline-inducible lentiviral expression system. A control HeLa cell line transduced with lentivirus carrying the CTRL was also included. Expression and similar localization of the transduced A151R constructs was confirmed by immunofluorescence assay (IFA) and immunoblotting (Fig. 3C and D, INPUT). The A151R-BAF interaction was subsequently assessed by coimmunoprecipitation (co-IP) using FLAG antibody-conjugated Dynabeads followed by immunoblotting. Consistent with the AlphaFold 3/FoldScript prediction, BAF was efficiently coimmunoprecipitated with A151R-WT (Fig. 3D, lane 2). In contrast, this interaction was markedly reduced in cells expressing either the A151R-A (Fig. 3D, lane 3) or A151R-K (Fig. 3D, lane 4) mutants, as BAF was not detected in the immunoprecipitates. From these results with A151R-K, we infer that residues Glu34 and Glu88 are critical for A151R-BAF complex formation, consistent with the AlphaFold 3 model. Data from A151R-A are also consistent with this interpretation but could be confounded by the lower expression of that mutant.

**Fig 4:**
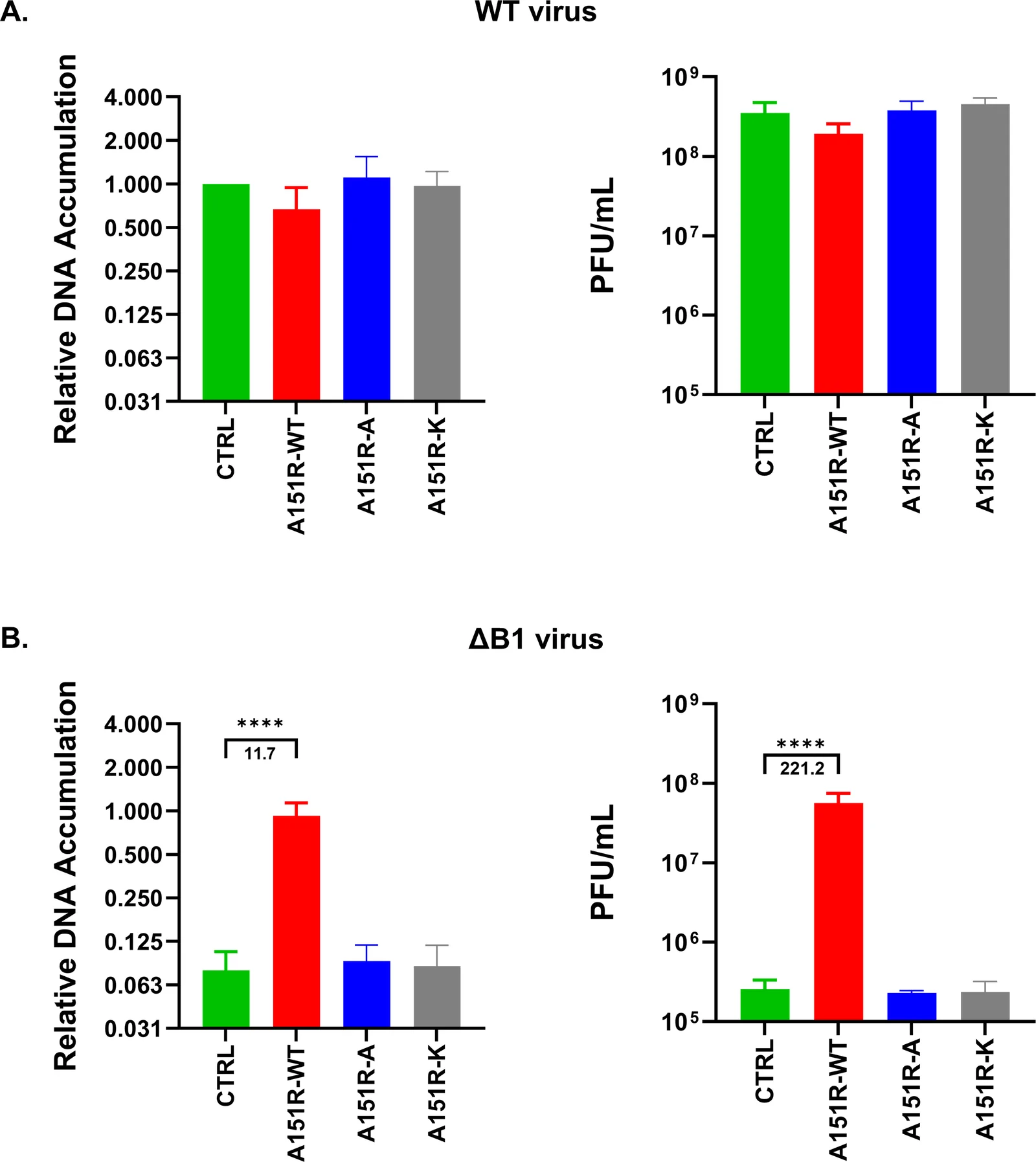
Impact of A151R-BAF interaction on WT and ΔB1 vaccinia virus replication. (**A and B**) DNA accumulation and viral yield of WT (**A**) or ΔB1 (**B**) vaccinia virus were quantified at 24 hpi in HeLa cells expressing FLAG-tagged wild-type A151R (A151R-WT), alanine substitution mutant (A151R-A), or lysine substitution mutant (A151R-K), or CTRL cells infected at an MOI of 3. Data are from more than five independent experimental replicates. ****, *P*  <  0.0001. Relative fold differences are given under each bracket.

We next assessed the functional consequence of disrupting this interaction during ΔB1 vaccinia virus infection. Cells were infected with either WT or ΔB1 vaccinia virus at a MOI of 3. At 24 h post-infection, the expression of A151R variants had no effects on WT vaccinia virus DNA accumulation (Fig. 4A, left) and progeny production (Fig. 4A, right). In contrast, A151R-WT expression significantly rescued ΔB1 vaccinia virus replication, as shown by increased viral DNA accumulation (11.7-fold) and progeny production (221.2-fold) (Fig. 4B). Notably, this effect was abolished when the A151R-BAF interaction was disrupted, as neither A151R mutant construct restored ΔB1 virus replication (Fig. 4B). Together, these results demonstrate that interaction between A151R and BAF is required for the promotion of BAF-sensitive virus replication. As both A151R-A and A151R-K mutants behaved identically and the A151R-K mutant was expressed at a level comparable to that of A151R-WT, only the A151R-K mutant was used in subsequent experiments.

### A151R binding to BAF impairs BAF’s DNA-binding activity

**Fig 5:**
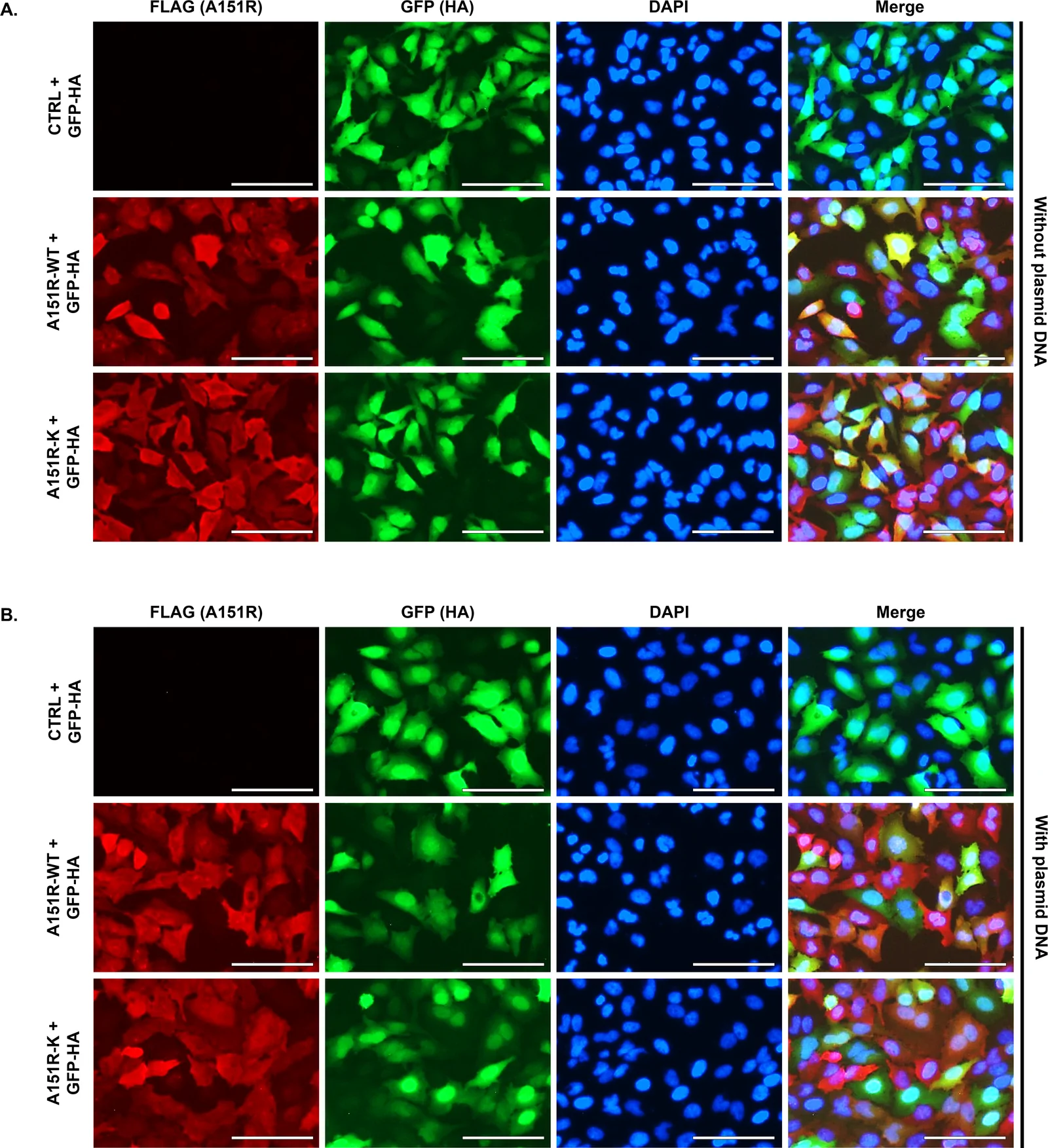
Localization of GFP-HA during plasmid DNA transfection. HeLa cells expressing FLAG-tagged A151R-WT or A151R-K, or CTRL cells, were transduced with GFP-HA-expressing lentivirus. Cells were incubated for 7 h in the presence of Lipofectamine 2000 alone (**A**) or transfected with 2 μg plasmid DNA using Lipofectamine 2000 (**B**), followed by immunofluorescence staining with anti-FLAG antibody (red). GFP signal (green) and nuclei counterstained with DAPI (blue) are shown. All images were acquired at 10× magnification from the same wells. Scale bar, 100 μm.

**Fig 6:**
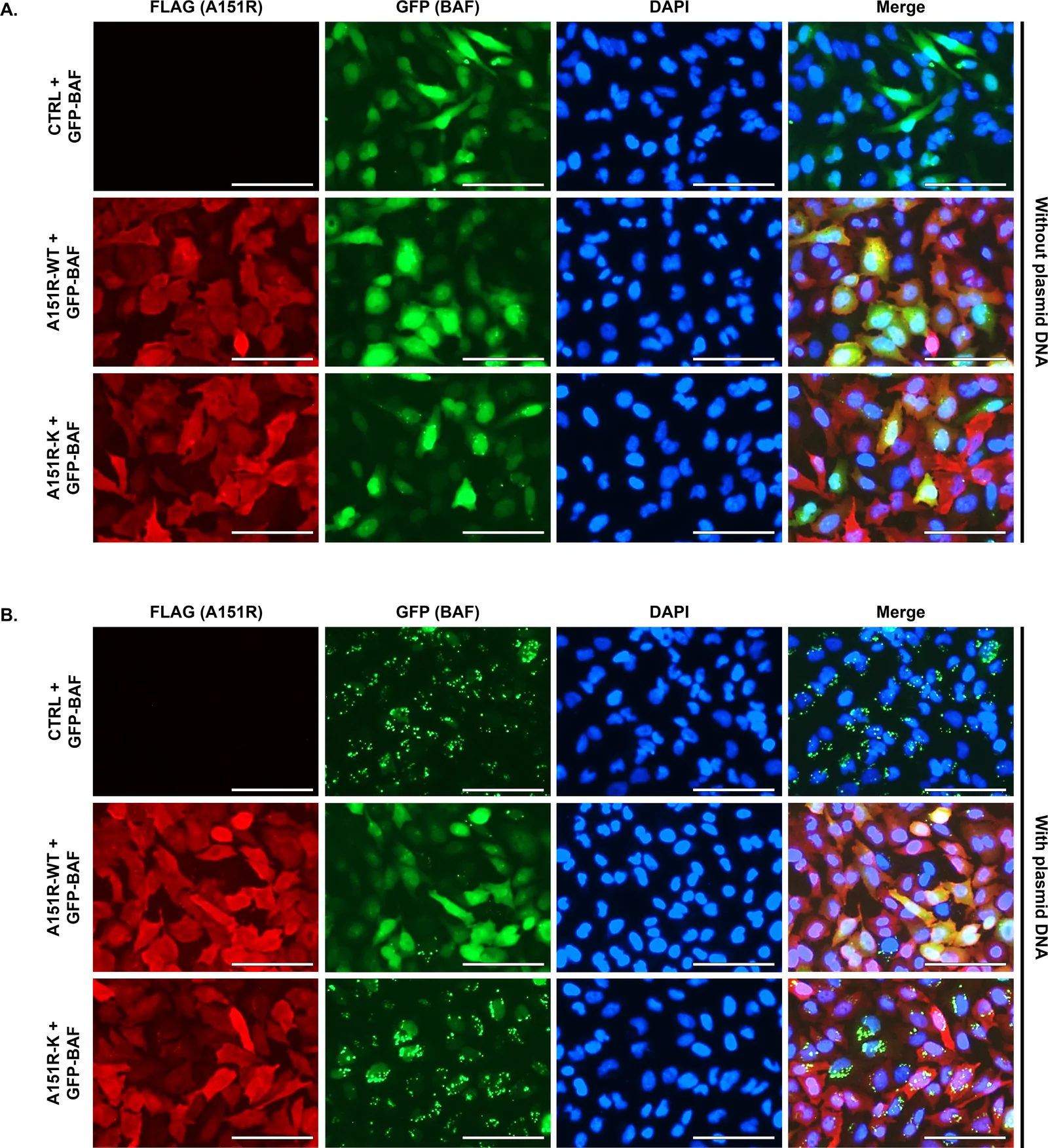
Localization of GFP-BAF during plasmid DNA transfection. HeLa cells expressing FLAG-tagged A151R-WT or A151R-K, or CTRL cells were transduced with GFP-BAF-expressing lentivirus. Cells were incubated for 7 h in the presence of Lipofectamine 2000 alone (**A**) or transfected with 2 μg plasmid DNA using Lipofectamine 2000 (**B**), followed by immunofluorescence staining with anti-FLAG antibody (red). GFP signal (green) and nuclei counterstained with DAPI (blue) are shown. All images were acquired at 10× magnification from the same wells. Scale bar, 100 μm.

**Fig 7:**
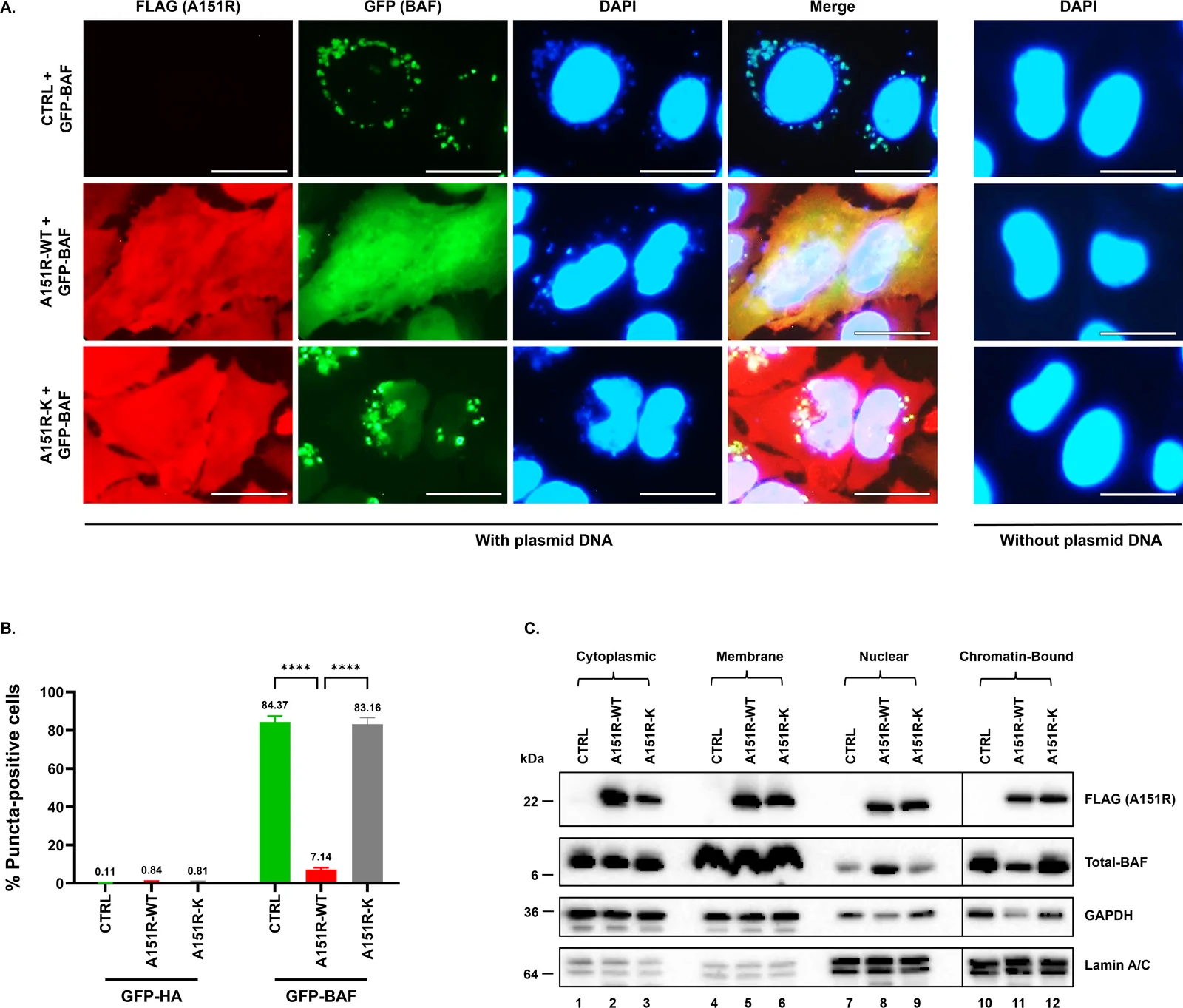
Effect of A151R on dsDNA-binding activity of BAF. HeLa cells expressing FLAG-tagged A151R-WT or A151R-K, or CTRL cells were transduced with GFP-BAF-expressing lentivirus. (**A**) Cells were transfected with 2 μg of plasmid DNA using Lipofectamine 2000 (with plasmid DNA panel) or treated with Lipofectamine 2000 alone (without plasmid DNA panel) and incubated for 7 h. Cells were then subjected to immunofluorescence staining with an anti-FLAG antibody (red). GFP fluorescence is shown in green, and nuclei were counterstained with DAPI (blue). All images were acquired at 40× magnification from the same wells. Scale bar, 25 μm. (**B**) The percentage of puncta-positive cells was quantified from four randomly selected fields captured at 10× magnification by IFA. Cells containing discrete GFP puncta were scored as puncta-positive cells. Percentage values are indicated above each bar. ****, *P* < 0.0001. (**C**) Immunoblot analysis of endogenous BAF in doxycycline-treated HeLa cells expressing FLAG-tagged A151R-WT, A151R-K, or CTRL cells following subcellular fractionation. GAPDH and lamin A/C were used as loading controls. Data are from more than three independent experimental replicates. A representative immunoblot is shown with lane numbers shown below each column.

BAF strongly inhibits vaccinia virus DNA replication, and its antiviral activity depends on its dsDNA-binding property (6, 8). Based on our AlphaFold 3 predictions (Fig. 1), A151R-interacting residues coincide with the dsDNA-binding region of BAF, suggesting that A151R targets the functional DNA-binding surface of BAF. Based on this, we hypothesized that A151R-BAF interaction promotes ΔB1 vaccinia virus replication by interfering with BAF-DNA binding. Plasmid DNA transfection has been used to assess BAF DNA-binding activity in the cytoplasm, as BAF efficiently relocalizes to transfected DNA and this binding has been confirmed by chromatin immunoprecipitation assay (8, 13). To test the impact of A151R on BAF-plasmid interaction, stable HeLa cell lines expressing N-terminal FLAG-tagged A151R-WT or A151R-K, or CTRL cells, were subsequently transduced with lentiviral constructs encoding either GFP-BAF or GFP tagged only with the HA epitope (GFP-HA) as a negative control. We next transfected the transduced cells with plasmid DNA using Lipofectamine 2000, with Lipofectamine 2000 alone serving as a negative control. At 7 h post-transfection, all cells expressing GFP-HA showed diffuse GFP signal throughout the cells, both in the absence (Fig. 5A) and presence (Fig. 5B) of plasmid DNA, with no evidence of aggregation or punctate structures, indicating that GFP localization was not affected by the presence of plasmid DNA. Similar results were observed in cells expressing GFP-BAF in the absence of plasmid DNA transfection (Fig. 6A). As expected, in cells transfected with plasmid DNA, GFP-BAF alone formed prominent punctate structures (Fig. 6B, first row), which colocalized with 4′,6-diamidino-2-phenylindole (DAPI) staining (Fig. 7A, first row), consistent with BAF binding to dsDNA. Interestingly, coexpression of A151R-WT resulted in a marked reduction in GFP-BAF puncta formation, with GFP-BAF instead exhibiting a diffuse distribution throughout both the nucleus and cytoplasm (Fig. 6B, second row, and Fig. 7A, second row), resembling the distribution observed in the absence of plasmid DNA transfection (Fig. 6A, second row). In contrast to A151R-WT, in the presence of A151R-K, GFP-BAF exhibited clear puncta formation (Fig. 6B, third row) and colocalization with plasmid DNA (Fig. 7A, third row) to levels comparable to cells expressing GFP-BAF alone (Fig. 6B, first row, and Fig. 7A, first row). Quantification further confirmed these observations, showing that A151R-WT significantly reduced the proportion of GFP-BAF puncta-positive cells from 84.37% in control cells to 7.14%, whereas upon A151R-K expression this proportion was 83.16%, comparable to control levels (Fig. 7B).

To complement these studies in another assay, we also investigated the subcellular localization of BAF, which is regulated to a considerable degree by its DNA-binding activity and association with genomic DNA (8, 13). Specifically, we therefore hypothesized that BAF localization may be altered in the presence or absence of A151R. To test this, subcellular fractionation was performed in stable HeLa cell lines expressing N-terminal FLAG-tagged A151R-WT or A151R-K, or CTRL cells, followed by immunoblotting. No differences in BAF levels were observed in the cytoplasmic and membrane fractions (Fig. 7C, lanes 1–6). Notably, A151R-WT reduced chromatin-bound BAF, as indicated by decreased signal in the chromatin fraction (Fig. 7C, lane 11) with a corresponding increase in the nuclear soluble fraction (Fig. 7C, lane 8) compared to the CTRL cells (Fig. 7C, lanes 7 and 10). In contrast, A151R-K had no effect on BAF distribution comparable to control cells, with chromatin-associated and nuclear soluble fractions resembling those observed under control conditions (Fig. 7C, lanes 9 and 12). Together, these results support a model in which A151R can interfere with nuclear BAF through its interaction, resulting in impaired dsDNA-binding activity, perhaps via steric hindrance of nucleic acid-binding residues, and consequently promote replication of an otherwise BAF-sensitive virus.

### A151R impairment of BAF is independent of phosphorylation but correlates with disrupted dimerization

**Fig 8:**
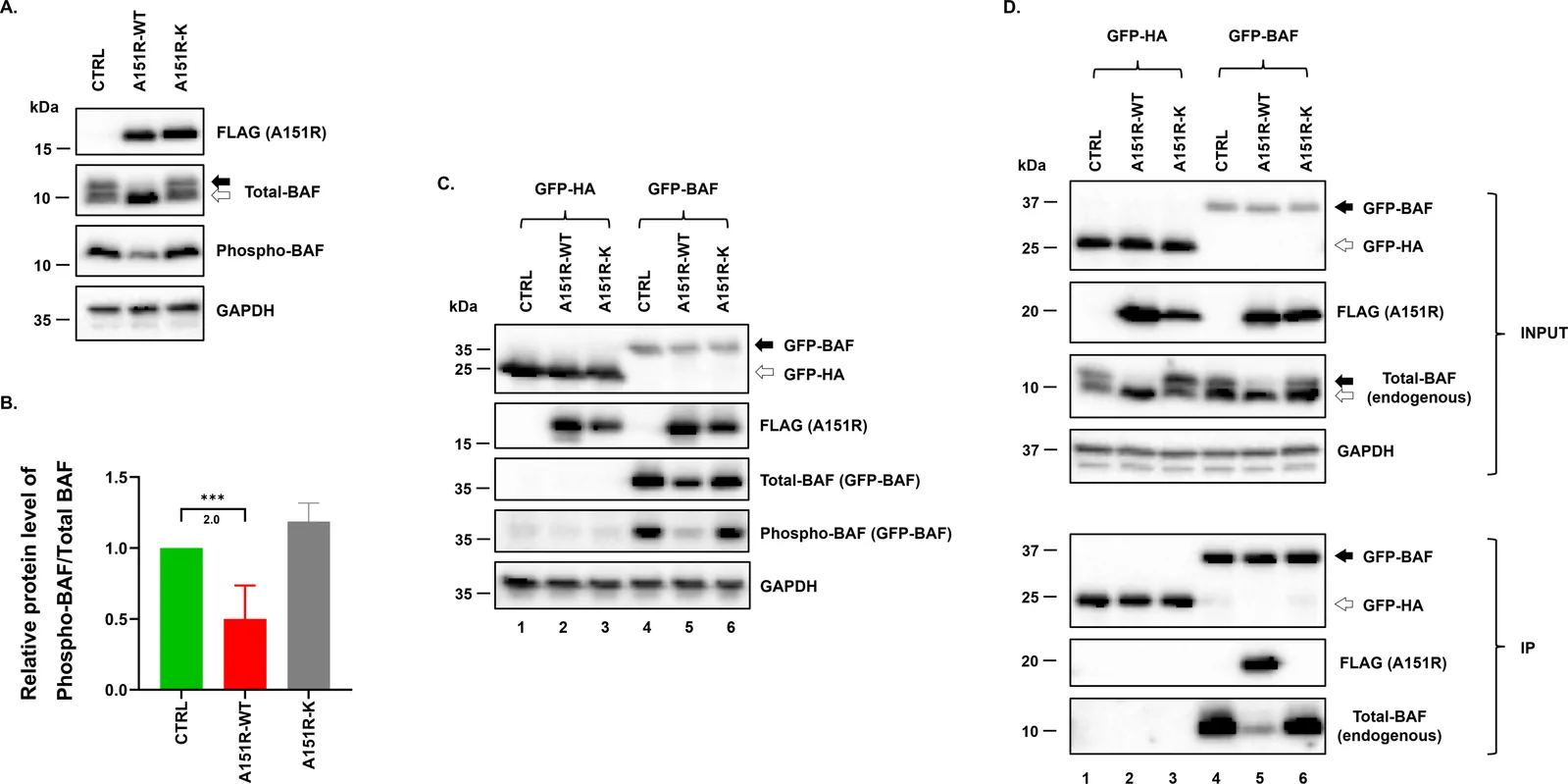
Impact of A151R on BAF phosphorylation and dimerization. (**A**) Immunoblot analysis of endogenous BAF in doxycycline-treated HeLa cells expressing FLAG-tagged A151R-WT, A151R-K, or CTRL cells. GAPDH was used as a loading control. (**B**) Relative protein levels of phospho-BAF/total BAF were quantified using ImageLab software (Bio-Rad) from the five independent replicates. The values for CTRL cells were normalized to 1. (**C**) Immunoblot analysis of GFP-BAF in doxycycline-treated HeLa cells expressing FLAG-tagged A151R-WT, A151R-K, or CTRL cells. GAPDH was used as a loading control. (**D**) Coimmunoprecipitation analysis of BAF dimerization. HeLa cells expressing FLAG-tagged A151R-WT or A151R-K, or CTRL cells were transduced with GFP-BAF-expressing lentivirus. Cell lysates from doxycycline-induced cells were immunoprecipitated using ChromoTek GFP-Trap magnetic agarose and analyzed by immunoblotting with antibodies against endogenous BAF. INPUT samples were included to confirm protein expression levels. GAPDH served as a loading control. The closed arrow indicates phosphorylated BAF, and the open arrow indicates unphosphorylated BAF in the indicated "total BAF” blots. Data are from more than three independent experimental replicates. A representative immunoblot is shown with lane numbers below each column. ***, *P* < 0.001.

We next sought to investigate how A151R impairs the dsDNA-binding activity of BAF in greater detail. BAF activity is dynamically regulated by reversible phosphorylation, which modulates its dsDNA-binding affinity and thereby controls its functions during mitosis and antiviral defense (5, 6, 8–12, 18–20). Accordingly, we hypothesized that A151R may in some way increase BAF phosphorylation, which inhibits BAF DNA binding as shown above, and thus, we examined BAF phosphorylation by Western blot analysis. BAF phosphorylation was monitored both by using a phospho-specific BAF antibody and using a “total” BAF antibody, which is known to detect phosphorylated and unphosphorylated BAF as two differentially migrating bands (6). Unexpectedly, expression of A151R-WT resulted in a marked reduction in phosphorylated BAF levels (Fig. 8A), with quantification showing an approximately twofold decrease relative to total BAF (Fig. 8B). In contrast, A151R-K had no detectable effect on BAF phosphorylation (Fig. 8A and B). Although distinct unphosphorylated and phosphorylated BAF species were not detected by anti-GFP immunoblotting in GFP-BAF-expressing cells (Fig. 8C), phosphorylated GFP-BAF was clearly detected in both CTRL and A151R-K-expressing cells (Fig. 8C, lanes 4 and 6). A reduction in phosphorylated GFP-BAF level was also observed in A151R-WT-expressing cells (Fig. 8C, lane 5) comparable to that of endogenous untagged BAF under the corresponding conditions (Fig. 8A). This observation indicates that GFP tagging does not alter BAF phosphorylation. Together, these results are again consistent with the possibility of steric hindrance of BAF in an A151R-bound complex. Specifically, A151R is predicted to directly engage Thr3 and Ser4 (Fig. 3A), the phosphorylation site of BAF, suggesting that A151R binding may mask those amino acids and thereby prevent its phosphorylation. Together with our prior data, these findings demonstrate that A151R promotes BAF-dependent viral replication independently of BAF phosphorylation.

Further investigating possible impacts of A151R on BAF, we also examined whether A151R affects BAF dimerization. BAF dimerization strongly impacts its affinity for DNA and is also necessary for its antipoxviral activity (8). To investigate whether A151R-BAF interaction affects GFP-BAF dimerization with endogenous untagged BAF, we next performed GFP-Trap coimmunoprecipitation followed by immunoblotting. Correlated with phosphorylation status in the cells without GFP-tagged proteins (Fig. 8A and B), the phosphorylation of endogenous BAF was decreased upon A151R expression (Fig. 8D; INPUT, lanes 2 and 5). No coprecipitation of either A151R or endogenous BAF was detected in cells expressing GFP-HA (Fig. 8D, lanes 1–3), indicating specificity of the assay. In cells expressing GFP-BAF, A151R-WT was efficiently coimmunoprecipitated (Fig. 8D, lane 5) with GFP-BAF, whereas no interaction was detected with the A151R-K mutant (Fig. 8D, lane 6), consistent with the FLAG-based co-IP results (Fig. 3C). Notably, coprecipitation of endogenous BAF was clearly reduced in cells expressing A151R-WT compared with CTRL cells (Fig. 8D; compare lane 5 to lane 4). In contrast, expression of A151R-K had no impact on endogenous BAF association with GFP-BAF compared to control conditions (Fig. 8D; compare lane 6 to lane 4). Together, these data indicate that A151R-BAF interaction also decreases BAF dimerization, providing further mechanistic explanation for the observed loss of dsDNA-binding activity and antiviral function.

### A151R reduces nuclear retention of BAF-MAAAQ

**Fig 9:**
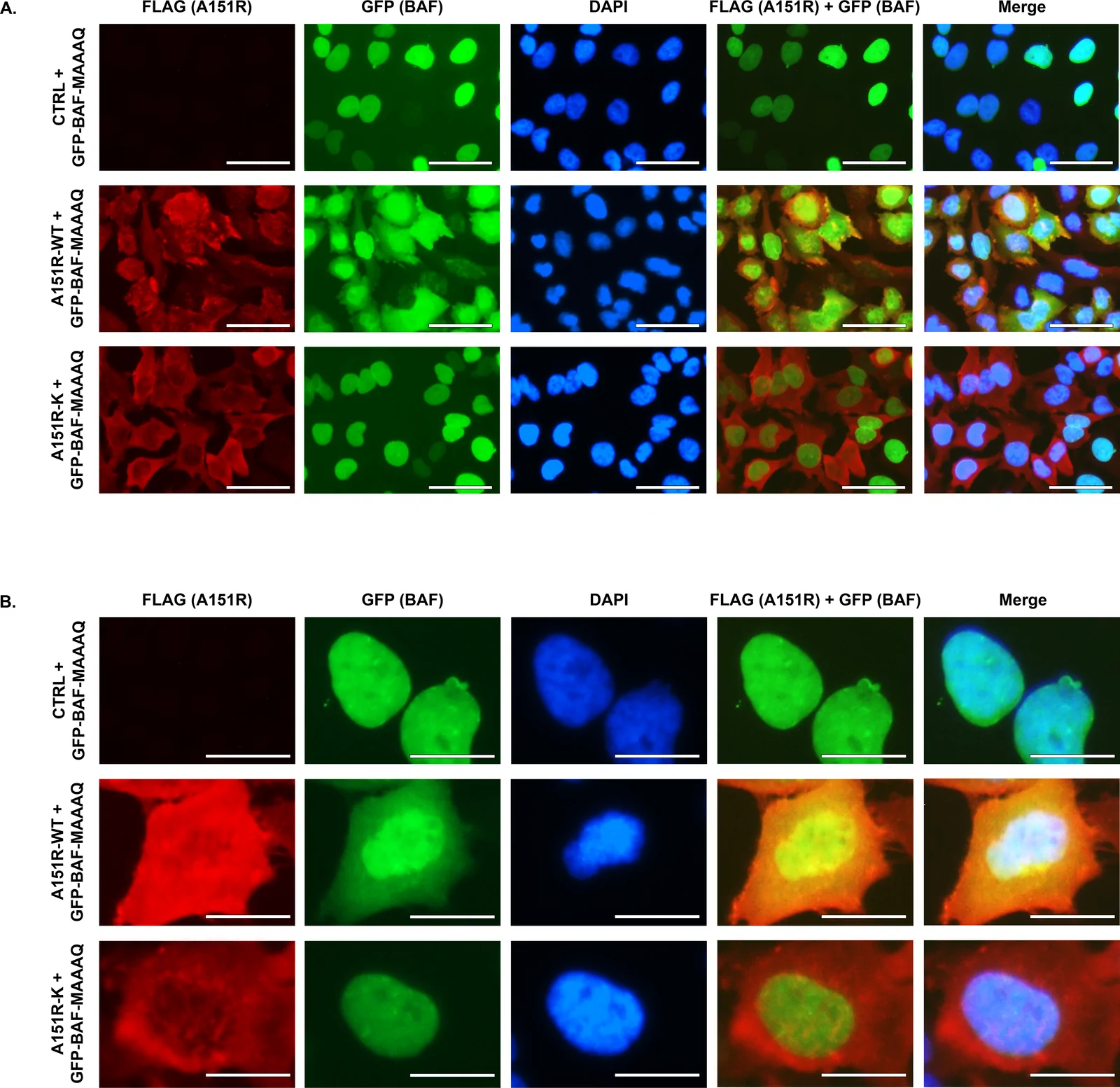
Effect of A151R on GFP-BAF-MAAAQ localization. (**A**) HeLa cells expressing FLAG-tagged A151R-WT or A151R-K, or CTRL cells were transduced with GFP-BAF-MAAAQ-expressing lentivirus. Protein localizations were analyzed by immunofluorescence staining with anti-FLAG antibody (red). GFP signal (green) and nuclei counterstained with DAPI (blue) are shown. All images were acquired at 40× magnification from the same wells. Scale bar, 50 μm. (**B**) Enlarged images from the same experiment highlighting subcellular localization. Scale bar, 20 μm.

To further investigate whether A151R can modulate BAF independently of its phosphorylation status, we utilized the previously characterized unphosphorylatable BAF mutant BAF-MAAAQ, in which Thr2, Thr3, and Ser4 are substituted with alanine (9, 13). BAF-MAAAQ has been shown to retain DNA-binding activity and exhibit enhanced chromatin association, resulting in predominant nuclear localization due to more constant genomic association than WT BAF (9, 13). Thus, stable HeLa cell lines expressing N-terminal FLAG-tagged A151R-WT or A151R-K, or CTRL cells, were transduced with lentiviral constructs encoding GFP-BAF-MAAAQ and the GFP-BAF-MAAAQ localization was analyzed by IFA (Fig. 9). Consistent with previous studies (9, 13), GFP-BAF-MAAAQ was found almost exclusively in the nucleus in CTRL cells (Fig. 9A and B, first row). In contrast, expression of A151R-WT resulted in marked redistribution of GFP-BAF-MAAAQ, with partial accumulation in the cytoplasm (Fig. 9A and B, second row). This effect was not observed in A151R-K-expressing cells, in which GFP-BAF-MAAAQ remained predominantly nuclear, similar to CTRL cells (Fig. 9A and B, third row). Together, these results demonstrate that A151R reduces the nuclear retention of unphosphorylated BAF and alters its subcellular localization despite the absence of regulatory phosphorylation sites. These findings further support a phosphorylation-independent mechanism by which A151R antagonizes BAF function.

## DISCUSSION

BAF is a cellular protein that binds exogenous DNA and acts as a host defense factor that can inhibit vaccinia virus DNA replication (5–7). BAF also modulates other pathways involved in maintenance of genomic integrity, including mitosis, DNA damage responses, and cGAS-mediated DNA sensing (2–7, 33–38). Importantly, each of these functions involves DNA binding by BAF, demonstrating the broad biological relevance of understanding how BAF DNA binding is regulated. In the case of cytoplasmic DNA viruses, numerous poxviruses encode a homolog of the vaccinia B1 kinase, which mimics cellular VRKs to disrupt BAF-DNA binding, thereby reducing viral DNA compaction and promoting viral replication (5, 6, 8, 18–20). In contrast, the ASFV also undergoes cytoplasmic replication but does not encode a B1-like kinase to inactivate BAF. However, the ASFV A151R protein was reported to interact with BAF (28) and has been implicated in ASFV replication, although the underlying mechanism remains unclear (26–28, 30, 39).

In this study, we identify a functional consequence of the A151R-BAF interaction on the dsDNA-binding activity of BAF. Structural modeling using AlphaFold and FoldScript revealed that A151R-interacting residues overlap with the dsDNA-binding region of BAF, suggesting that A151R targets the functional DNA-binding surface for steric hindrance. This prediction was supported by coimmunoprecipitation assays, which confirmed A151R-BAF complex formation consistent with a previous report (28) and showed that substitutions at residues 34 and 88 of A151R abrogated the interaction. Functionally, A151R had no effect on WT virus replication in the cell lines used in this study, where BAF restriction is efficiently antagonized by the viral B1 kinase (5, 6, 8, 16, 18–21). In contrast, A151R promoted DNA replication and progeny production of a BAF-restricted ∆B1 virus in both HeLa and HAP1-VRK2KO cells, indicating that A151R compensates for the loss of B1-mediated antagonism of BAF to support viral DNA replication. This phenotype was abolished upon disruption of the A151R-BAF interaction, supporting a direct requirement for complex formation. Consistent with the antiviral role of BAF, A151R expression markedly impaired BAF DNA-binding activity, providing a mechanistic basis for enhanced viral replication. Collectively, these data demonstrate that A151R promotes virus replication by functionally targeting the BAF DNA-binding interface.

BAF activity is regulated by reversible phosphorylation and dephosphorylation, which controls its DNA-binding affinity (9–12) both in the nucleus and cytoplasm. Phosphorylated BAF dissociates from dsDNA, whereas the dephosphorylated form binds DNA with high affinity (5, 9–12). Unexpectedly, A151R expression resulted in accumulation of dephosphorylated BAF, suggesting interference with phosphorylation site accessibility at Thr2, Thr3, and Ser4 (6, 9, 13). Our studies reveal that despite the accumulation of dephosphorylated BAF, BAF DNA-binding activity was not enhanced if A151R is present, as shown by reduced chromatin association and impaired DNA binding in functional assays. These findings indicate that phosphorylation status alone is insufficient to explain BAF activity under these conditions. To further dissect this discrepancy, we examined the unphosphorylatable BAF mutant BAF-MAAAQ, which is predominantly nuclear and exhibits strong chromatin association (13). In the presence of A151R, BAF-MAAAQ was redistributed from the nucleus and partially relocalized to the cytoplasm, supporting a phosphorylation-independent mechanism of BAF regulation. Importantly, this effect required the A151R-BAF interaction. The mechanism underlying this redistribution remains unclear and will require further investigation. In addition to its effect on BAF localization, A151R also reduced BAF dimerization, an interaction required for high-affinity DNA binding and antiviral activity (2, 8). Reduced BAF dimerization was accompanied by loss of chromatin-associated BAF and impaired DNA binding, further supporting a role for BAF dimerization in its functional activity, consistent with previous studies demonstrating that the BAF-G47E mutant, which disrupts homodimerization, exhibits markedly reduced DNA binding and loss of antiviral activity (8). Together, these results support a model in which A151R impairs BAF function by obscuring both its DNA-binding site as well as its phosphorylatable N-terminal tail. The reduction in BAF dimerization triggered by A151R is perhaps caused by an interaction not predicted by AlphaFold or indirectly resulting from loss of DNA binding that would stabilize homodimer formation.

While the studies presented here in the context of vaccinia infection provide novel insights into A151R-BAF signaling, the roles these proteins play during ASFV infection are likely more complex. Specifically, BAF depletion has been reported to reduce ASFV titers (28), suggesting BAF also has a yet-to-be-discovered proviral role for this pathogen. While this may appear inconsistent with the antiviral role of BAF described in our previous studies on vaccinia virus, we postulate that it reflects the unique complexity of ASFV infection, in which viral and host factors may differentially influence BAF-associated cellular functions, depending on their subcellular location. As an example of this unique complexity, the ASFV life cycle includes both a nuclear and cytoplasmic phase (40–44), while poxviruses replicate their genomes solely in the cytoplasm. BAF mediates proviral signaling during herpesvirus infection (45), and it may be acting in the same manner for part of the ASFV life cycle as well. Clearly, further studies will be required to dissect the full spectrum of viral and host determinants that modulate BAF function in the context of ASFV infection as well as whether as-yet untested cell type-specific regulation of A151R may occur.

In summary, this study identifies a previously unrecognized mechanism by which the ASFV A151R protein promotes replication of a BAF-inhibited virus by targeting the BAF dsDNA-binding interface. A151R acts as a viral antagonist of BAF, impairing its DNA-binding activity and antiviral function independently of phosphorylation status. This inhibition correlates with reduced BAF dimerization and loss of chromatin association, indicating that A151R disrupts the structural and functional integrity of BAF required for antiviral activity. Importantly, these effects are strictly dependent on the A151R-BAF interaction, as disruption of the binding interface abolishes all observed phenotypes. Together, these findings reveal a novel phosphorylation-independent strategy for BAF antagonism and establish A151R as a key ASFV factor that counteracts BAF activity to promote viral replication. Further investigation in porcine cell systems will provide additional insight into the role of BAF during ASFV infection and the contribution of A151R-mediated BAF regulation in the natural host context. Collectively, regulation of BAF likely represents a common challenge for cytoplasmic DNA viruses, suggesting that additional viral mechanisms for controlling BAF remain to be discovered.

## MATERIALS AND METHODS

### Protein interaction prediction

Human BAF (UniProtKB: O75531) and porcine BAF (UniProtKB: F1RU33) sequences were retrieved and aligned using BioEdit software (version 7.7.1) to determine sequence identity. Structural modeling of BAF-DNA and BAF-A151R interactions was performed using AlphaFold 3 (29). Protein interaction interfaces were analyzed using FoldScript (31). Model quality scores, including predicted template modeling (pTM) and ipTM, were recorded as provided by AlphaFold 3 output generated using default settings. Structural comparisons between AlphaFold-produced models and existing crystal structures of A151R or BAF were performed using PyMOL Software (The PyMOL Molecular Graphics System, version 2.5.5; Schrödinger, LLC). The RMSD was calculated by aligning the monomeric structure of either BAF or A151R obtained from the Protein Data Bank (RCSB.org) with the AlphaFold produced structure.

### Cell culture

Human kidney 293T cells were purchased from ATCC. Human cervix epithelial adenocarcinoma HeLa cells were purchased and obtained directly from the ATCC. African green monkey kidney CV1 cells were obtained from Invitrogen Life Technologies. These cell lines were maintained in high-glucose Dulbecco’s modified Eagle’s medium (high glucose, Cytiva HyClone) supplemented with 10% fetal bovine serum (FBS, Atlanta Biologicals) and penicillin-streptomycin. Human near-haploid fibroblast HAP1 parental and vaccinia virus-related kinase 2 knockout cells were obtained from Horizon Genomics and maintained in Iscove’s modified Dulbecco’s medium (Cytiva HyClone) supplemented with 10% FBS (Atlanta Biologicals) and penicillin-streptomycin. All cells were incubated at 37°C in a 5% CO_2_ atmosphere.

### Plasmid construction

Each 3XFLAG-tagged A151R wild-type (A151R-WT) and mutant construct (A151R-A and A151R-K) in the pTWISTcDNA3.1 vector were purchased from Thermo Fisher Scientific. The A151R-WT, A151R-A, or A151R-K open reading frame was digested with NheI and BamHI and ligated into the pCW57-PURO-MCS1-2A-MCS2 vector to generate pCW57-PURO-3XFLAG-A151R-WT, pCW57-PURO-3XFLAG-A151R-A, or pCW57-PURO-3XFLAG-A151R-K plasmids, respectively. The pCW57-MCS1-2A-MCS2 vector was a gift from Adam Karpf (Addgene plasmid #71782). The pHAGE-Hyg-GFP-HA and pHAGE-Hyg-GFP-BAF were constructed using standard cloning techniques from the previously described pAcGFP and pAcGFP-BAF plasmids (9). The pHAGE-Hyg-GFP-MAAAQ was generated from the pHAGE-Hyg-GFP-BAF plasmid using the Q5 Site-Directed Mutagenesis Kit (New England Biolabs) with BAF-MAAAQ-F primer (5′-CCGCCCAAAAGCACCGAGACTTCGTG-3′) together with pHAGE-GFP-R primer (5′-CTGCCATAAGCTTGAGCTCGAGATC-3′). Plasmids were confirmed by DNA sequencing.

### Lentivirus production

The lentiviruses encapsulating pCW57-PURO-MCS1-2A-MCS2 (control [CTRL]), pCW57-PURO-3XFLAG-A151R-WT, pCW57-PURO-3XFLAG-A151R-A, pCW57-PURO-3XFLAG-A151R-K, pHAGE-Hyg-GFP-HA, pHAGE-Hyg-GFP-BAF, or pHAGE-Hyg-GFP-MAAAQ were produced in 293T cells. Cells were transfected with the individual plasmid, along with pVSVG and psPAX2 helper plasmids, using Lipofectamine 2000 (Thermo Fisher Scientific) (46). Fresh media supplemented with 5 mM sodium butyrate (EMD Millipore Corp.) and 1× GlutaMAX (Gibco) were added to cells 16 h post-transfection. At 24 h post-transfection, fresh media supplemented with 10 mM HEPES (Thermo Fisher Scientific) and 1× GlutaMAX (Gibco) were added to the cells. Virus in the supernatant was collected at 48 h post-transfection and filtered through a 0.45 μm syringe. Polybrene (Thermo Fisher Scientific) was added to lentivirus at 10 μg/mL and used immediately to generate the stably transduced cell lines.

### Generation of stably transduced cell lines

To generate stably transduced cell lines, HeLa, HAP1, or HAP1-VRK2KO cells were seeded at 25% confluence in 35 mm dishes. After 24 h, the culture medium was replaced with 0.8 mL of lentiviral supernatant encapsulated pCW57-PURO-MCS1-2A-MCS2 (CTRL), pCW57-PURO-3XFLAG-A151R-WT, pCW57-PURO-3XFLAG-A151R-A, or pCW57-PURO-3XFLAG-A151R-K and incubated for 16 h. The cell supernatant was then removed and replaced with fresh growth medium, followed by an additional 24 h incubation. To select for stable lentiviral integration, cells were passaged in a medium containing 0.5 µg/mL puromycin for HAP1 and HAP1-VRK2KO cells or 1 µg/mL puromycin for HeLa cells. After selection, the cells were induced for 6 or 24 h with 0.5 µg/mL doxycycline to induce protein expression. Protein expression was confirmed by immunoblotting using a mouse anti-FLAG M2 (Sigma) antibody. The HeLa cell lines expressing 3XFLAG-A151R-WT or 3XFLAG-A151R-K, or CTRL cells, were subsequently transduced with lentiviral constructs encoding GFP-HA (negative control), GFP-BAF, or GFP-BAF-MAAAQ as described above. To select for stably transduced cells, cells were passaged in medium containing 1 μg/mL puromycin and 200 μg/mL hygromycin. Protein expression was confirmed by immunoblotting using mouse anti-FLAG (Sigma, F1804) and anti-GFP (Cell Signaling Technology, 2956S) antibodies.

### Small interfering RNA depletion

siVRK2 and non-targeting control (siCTRL) were previously validated and obtained from Dharmacon. The small interfering RNA (siRNA) sequences were as follows: siVRK2 (5′-CACAAUAGGUUAAUCGAAAUU-3′) and siCTRL (5′-CAGUCGCGUUUGCGACUGGUU-3′). Cells were transfected with 100 nM siRNA using Lipofectamine RNAiMAX (Life Technologies) according to the manufacturer’s instructions. Protein depletion was measured by immunoblot analysis at 72 h post-transfection.

### Viruses and viral infections

WT and B1-deleted virus (ΔB1) WR strain vaccinia viruses purified as described (21) were used in this study. For vaccinia viral DNA accumulation and viral titer determination, confluent monolayers of cells were infected with WT or ΔB1 virus at an MOI of 3 PFU per cell at 37°C for 24 h. Cells were harvested and divided into two equal aliquots for viral DNA accumulation and viral yield assays. For viral yield assays, following cell harvest, cells were pelleted and resuspended in 200 µL of 10 mM Tris (pH 9). Virus samples were freeze-thawed three times and titrated on complementing CV1B1 cells produced as previously described (21). Cells were fixed and stained at 72 h post-infection.

### DNA purification and quantitative PCR

Vaccinia viral DNA was extracted using the E.Z.N.A. Tissue DNA Kit (Omega Bio-tek) according to the manufacturer’s protocol. Quantitative PCR (qPCR) was performed using iTaq Universal SYBR Green Supermix (Bio-Rad) with specific primers targeting the vaccinia virus hemagglutinin (HA) gene: HA_Fwd (5′-CATCATCTGGAATTGTCACTACTAAA-3′) and HA_Rev (5′-ACGGCCGACAATTATAATTAATGC-3′) primers as previously described (13). Serial dilutions of vaccinia DNA were included in each qPCR run to generate a standard curve and determine the PCR efficiency of the primer set. Each qPCR reaction was performed using approximately 10 ng of DNA and 10 μM of each primer.

### Subcellular fractionation assay

Cells were fractionated into soluble cytoplasmic, membrane, soluble nuclear, and chromatin-bound nuclear fractions using a subcellular protein fractionation kit for cultured cells (Thermo Scientific) according to the manufacturer’s instructions, with the addition of phosphatase inhibitors. Glyceraldehyde-3-phosphate dehydrogenase (GAPDH) was used as a predominant cytosolic and minor membrane-associated protein control. Lamin A/C was used as a nuclear and chromatin-associated protein control. Samples were analyzed by immunoblotting.

### Protein coimmunoprecipitations

To determine the protein interaction, confluent monolayers of cells in 10 cm^2^ dishes were harvested in cold Dulbecco’s phosphate buffered saline (Cytiva HyClone) and pelleted. Cells were lysed with a lysis buffer containing 50 mM Tris-HCl (pH 7.4), 150 mM NaCl, and 1% Triton X-100, and then supplemented with protease and phosphatase inhibitors and nuclease (Pierce Universal Nuclease). Lysates were clarified following centrifugation, and 3XFLAG-A151R complexes were immunoprecipitated using Dynabead Protein G magnetic beads conjugated to mouse anti-FLAG antibodies (Sigma, F1804). GFP complexes were immunoprecipitated using ChromoTek GFP-Trap Magnetic Agarose (ProteinTech). Complexes were eluted in SDS sample buffer with protease and phosphatase inhibitors by incubating at 37°C for 24 h. Samples were evaluated by immunoblotting.

### Immunoblotting

To assess protein expression, cells were harvested, pelleted, and suspended in an SDS sample buffer supplemented with protease and phosphatase inhibitors and nuclease (Pierce Universal Nuclease). Whole-cell lysates were resolved on 18% SDS-PAGE gels and transferred to polyvinylidene difluoride membrane (Bio-Rad). The primary antibodies used are as follows: mouse anti-FLAG (1:3,000; Sigma, F1804), rabbit anti-FLAG (1:1,000; Thermo Fisher Scientific, 740001), mouse anti-VRK2 (1:500; Santa Cruz Biotechnology; sc-365199), rabbit anti-GAPDH (1:1,000; Santa Cruz Biotechnology; sc-25778), rabbit antitotal BAF (1:500, custom), rabbit antiphosphorylated BAF (1:100, custom), rabbit anti-L4 (1:2,000; custom), rabbit anti-GFP (1:2,000; Cell Signaling Technology, 2956S), and mouse antilamin A/C (1:1,000; Cell Signaling Technology, 4777S)

### Plasmid transfection

Cells were seeded at 50% confluence on the day of transfection and transfected with 2 μg of pcDNA3.1(+) plasmid using Lipofectamine 2000 (Invitrogen) according to the manufacturer’s instructions. Cells were incubated for 7 h prior to fixation for immunofluorescence assays.

### Immunofluorescence assays

Cells were fixed in 4% paraformaldehyde in PBS and permeabilized with 0.2% Triton X-100 in PBS for 5 min at room temperature. The primary antibodies used were mouse anti-FLAG (1:400; Sigma, F1804), followed by Alexa Fluor 594-conjugated secondary antibodies against FLAG. Nuclei were counterstained with DAPI. Fluorescence images were acquired on an inverted fluorescence microscope (the EVOS FL Auto Imaging System).

### Statistics

All experiments were performed at least in triplicate, and graphed data represent the mean values of all experimental replicates. Error bars indicate 1 standard deviation from the mean. Statistical significance was assessed using one- or two-way analysis of variance or multiple *t*-tests, as appropriate, using GraphPad Prism (version 11.0.2) for Windows.

## Data Availability

All unique reagents generated in this study are available from the lead contact. All other data supporting the findings of this study are contained within the article.

## References

[1] Lee MS, Craigie R. 1998. A previously unidentified host protein protects retroviral DNA from autointegration. Proc Natl Acad Sci USA 95:1528–1533. doi:10.1073/pnas.95.4.1528 PMC19075 9465049

[2] Bradley CM, Ronning DR, Ghirlando R, Craigie R, Dyda F. 2005. Structural basis for DNA bridging by barrier-to-autointegration factor. Nat Struct Mol Biol 12:935–936. doi:10.1038/nsmb989 16155580

[3] Cai M, Huang Y, Zheng R, Wei SQ, Ghirlando R, Lee MS, Craigie R, Gronenborn AM, Clore GM. 1998. Solution structure of the cellular factor BAF responsible for protecting retroviral DNA from autointegration. Nat Struct Biol 5:903–909. doi:10.1038/2345 9783751

[4] Umland TC, Wei SQ, Craigie R, Davies DR. 2000. Structural basis of DNA bridging by barrier-to-autointegration factor. Biochemistry 39:9130–9138. doi:10.1021/bi000572w 10924106

[5] Ibrahim N, Wicklund A, Jamin A, Wiebe MS. 2013. Barrier to autointegration factor (BAF) inhibits vaccinia virus intermediate transcription in the absence of the viral B1 kinase. Virology 444:363–373. doi:10.1016/j.virol.2013.07.002 PMC3755115 23891157

[6] Wiebe MS, Traktman P. 2007. Poxviral B1 kinase overcomes barrier to autointegration factor, a host defense against virus replication. Cell Host Microbe 1:187–197. doi:10.1016/j.chom.2007.03.007 PMC1978190 18005698

[7] Saeng-Chuto K, Wang Z, Krueger AC, Bargeron K, Wiebe MS. 2025. A novel antiviral role of ankyrin repeat and LEM domain-containing 2 (ANKLE2) in restricting vaccinia virus through barrier to autointegration factor (BAF). J Virol 99:e01549-25. doi:10.1128/jvi.01549-25 PMC12724269 41251347

[8] Ibrahim N, Wicklund A, Wiebe MS. 2011. Molecular characterization of the host defense activity of the barrier to autointegration factor against vaccinia virus. J Virol 85:11588–11600. doi:10.1128/JVI.00641-11 PMC3209281 21880762

[9] Nichols RJ, Wiebe MS, Traktman P. 2006. The vaccinia-related kinases phosphorylate the N’ terminus of BAF, regulating its interaction with DNA and its retention in the nucleus. Mol Biol Cell 17:2451–2464. doi:10.1091/mbc.e05-12-1179 PMC1446082 16495336

[10] Li J, Wang X, Jordana L, Bonneil É, Ginestet V, Ahmed M, Bourouh M, Pascariu CM, Schmeing TM, Thibault P, Archambault V. 2025. Mechanisms of PP2A-Ankle2 dependent nuclear reassembly after mitosis. eLife 13:RP104233. doi:10.7554/eLife.104233 PMC11835388 39964262

[11] Asencio C, Davidson IF, Santarella-Mellwig R, Ly-Hartig TBN, Mall M, Wallenfang MR, Mattaj IW, Gorjánácz M. 2012. Coordination of kinase and phosphatase activities by Lem4 enables nuclear envelope reassembly during mitosis. Cell 150:122–135. doi:10.1016/j.cell.2012.04.043 22770216

[12] Li J, Jordana L, Mehsen H, Wang X, Archambault V. 2024. Nuclear reassembly defects after mitosis trigger apoptotic and p53-dependent safeguard mechanisms in *Drosophila*. PLoS Biol 22:e3002780. doi:10.1371/journal.pbio.3002780 PMC11379398 39186808

[13] Jamin A, Wicklund A, Wiebe MS. 2014. Cell- and virus-mediated regulation of the barrier-to-autointegration factor’s phosphorylation state controls its DNA binding, dimerization, subcellular localization, and antipoxviral activity. J Virol 88:5342–5355. doi:10.1128/JVI.00427-14 PMC4019112 24600006

[14] Yang Z, Cao S, Martens CA, Porcella SF, Xie Z, Ma M, Shen B, Moss B. 2015. Deciphering poxvirus gene expression by RNA sequencing and ribosome profiling. J Virol 89:6874–6886. doi:10.1128/JVI.00528-15 PMC4468498 25903347

[15] Skoko D, Li M, Huang Y, Mizuuchi M, Cai M, Bradley CM, Pease PJ, Xiao B, Marko JF, Craigie R, Mizuuchi K. 2009. Barrier-to-autointegration factor (BAF) condenses DNA by looping. Proc Natl Acad Sci USA 106:16610–16615. doi:10.1073/pnas.0909077106 PMC2748091 19805345

[16] Rico AB, Linville AC, Olson AT, Wang Z, Wiebe MS. 2021. The vaccinia virus B12 pseudokinase represses viral replication via interaction with the cellular kinase VRK1 and activation of the antiviral effector BAF. J Virol 95:e02114-20. doi:10.1128/JVI.02114-20 PMC7925102 33177193

[17] Sears RM, Roux KJ. 2020. Diverse cellular functions of barrier-to-autointegration factor and its roles in disease. J Cell Sci 133:jcs246546. doi:10.1242/jcs.246546 PMC7449794 32817163

[18] Nezu J, Oku A, Jones MH, Shimane M. 1997. Identification of two novel human putative serine/threonine kinases, VRK1 and VRK2, with structural similarity to vaccinia virus B1R kinase. Genomics 45:327–331. doi:10.1006/geno.1997.4938 9344656

[19] Nichols RJ, Traktman P. 2004. Characterization of three paralogous members of the mammalian vaccinia related kinase family. J Biol Chem 279:7934–7946. doi:10.1074/jbc.M310813200 14645249

[20] Traktman P, Anderson MK, Rempel RE. 1989. Vaccinia virus encodes an essential gene with strong homology to protein kinases. J Biol Chem 264:21458–21461. doi:10.1016/S0021-9258(20)88203-9 2600076

[21] Olson AT, Rico AB, Wang Z, Delhon G, Wiebe MS. 2017. Deletion of the vaccinia virus B1 kinase reveals essential functions of this enzyme complemented partly by the homologous cellular kinase VRK2. J Virol 91:e00635-17. doi:10.1128/JVI.00635-17 PMC5512240 28515294

[22] Dixon LK, Chapman DAG, Netherton CL, Upton C. 2013. African swine fever virus replication and genomics. Virus Res 173:3–14. doi:10.1016/j.virusres.2012.10.020 23142553

[23] Yáñez RJ, Rodríguez JM, Nogal ML, Yuste L, Enríquez C, Rodriguez JF, Viñuela E. 1995. Analysis of the complete nucleotide sequence of African swine fever virus. Virology 208:249–278. doi:10.1006/viro.1995.1149 11831707

[24] Chapman DAG, Tcherepanov V, Upton C, Dixon LK. 2008. Comparison of the genome sequences of non-pathogenic and pathogenic African swine fever virus isolates. J Gen Virol 89:397–408. doi:10.1099/vir.0.83343-0 18198370

[25] Weng W, Wang H, Ye M, Hu D, Wu J, Qu Y, Gao P, Zhang Y, Zhou L, Ge X, Guo X, Han J, Yang H. 2025. Revisiting the early event of African swine fever virus DNA replication. J Virol 99:e0058425. doi:10.1128/jvi.00584-25 PMC12282160 40444991

[26] Ramirez-Medina E, Vuono E, Pruitt S, Rai A, Espinoza N, Valladares A, Spinard E, Silva E, Velazquez-Salinas L, Gladue DP, Borca MV. 2022. ASFV Gene A151R Is involved in the process of virulence in domestic swine. Viruses 14:1834. doi:10.3390/v14081834 PMC9413758 36016456

[27] Keita D, Heath L, Albina E. 2010. Control of African swine fever virus replication by small interfering RNA targeting the A151R and VP72 genes. Antivir Ther 15:727–736. doi:10.3851/IMP1593 PMC3101891 20710054

[28] Dupré J, Dolata KM, Pei G, Molouki A, Goatley LC, Küchler R, Soh TK, Bosse JB, Fablet A, Le Dimna M, Karadjian G, Hirchaud E, Netherton CL, Dixon LK, Reis AL, Vitour D, Le Potier M-F, Karger A, Caignard G. 2025. Exploring virus-host interactions through combined proteomic approaches Identifies BANF1 as a new essential factor for African swine fever virus. Mol Cell Proteomics 24:101038. doi:10.1016/j.mcpro.2025.101038 PMC12398270 40707000

[29] Abramson J, Adler J, Dunger J, Evans R, Green T, Pritzel A, Ronneberger O, Willmore L, Ballard AJ, Bambrick J, et al.. 2024. Accurate structure prediction of biomolecular interactions with AlphaFold 3. Nature 630:493–500. doi:10.1038/s41586-024-07487-w PMC11168924 38718835

[30] Huang JW, Niu D, Liu K, Wang Q, Ma L, Chen CC, Zhang L, Liu W, Zhou S, Min J, Wu S, Yang Y, Guo RT. 2020. Structure basis of non-structural protein pA151R from African swine fever virus. Biochem Biophys Res Commun 532:108–113. doi:10.1016/j.bbrc.2020.08.011 32828542

[31] Robert X, Guillon C, Gouet P. 2025. FoldScript: a web server for the efficient analysis of AI-generated 3D protein models. Nucleic Acids Res 53:W277–W282. doi:10.1093/nar/gkaf326 PMC12230654 40276967

[32] Rico AB, Wang Z, Olson AT, Linville AC, Bullard BL, Weaver EA, Jones C, Wiebe MS. 2019. The vaccinia virus (VACV) B1 and cellular VRK2 kinases promote VACV replication factory formation through phosphorylation-dependent inhibition of VACV B12. J Virol 93:e00855-19. doi:10.1128/JVI.00855-19 PMC6798115 31341052

[33] Ma H, Qian W, Bambouskova M, Collins PL, Porter SI, Byrum AK, Zhang R, Artyomov M, Oltz EM, Mosammaparast N, Miner JJ, Diamond MS. 2020. barrier-to-autointegration factor 1 protects against a basal cGAS-STING response. mBio 11:e00136-20. doi:10.1128/mBio.00136-20 PMC7064753 32156810

[34] Linville AC, Rico AB, Teague H, Binsted LE, Smith GL, Albarnaz JD, Wiebe MS. 2022. Dysregulation of cellular VRK1, BAF, and innate immune signaling by the vaccinia virus B12 pseudokinase. J Virol 96:e00398-22. doi:10.1128/jvi.00398-22 PMC9175622 35543552

[35] Halfmann CT, Sears RM, Katiyar A, Busselman BW, Aman LK, Zhang Q, O’Bryan CS, Angelini TE, Lele TP, Roux KJ. 2019. Repair of nuclear ruptures requires barrier-to-autointegration factor. J Cell Biol 218:2136–2149. doi:10.1083/jcb.201901116 PMC6605789 31147383

[36] Cai M, Huang Y, Ghirlando R, Wilson KL, Craigie R, Clore GM. 2001. Solution structure of the constant region of nuclear envelope protein LAP2 reveals two LEM-domain structures: one binds BAF and the other binds DNA. EMBO J 20:4399–4407. doi:10.1093/emboj/20.16.4399 PMC125263 11500367

[37] Segura-Totten M, Kowalski AK, Craigie R, Wilson KL. 2002. Barrier-to-autointegration factor: major roles in chromatin decondensation and nuclear assembly. J Cell Biol 158:475–485. doi:10.1083/jcb.200202019 PMC2173821 12163470

[38] Young AM, Gunn AL, Hatch EM. 2020. BAF facilitates interphase nuclear membrane repair through recruitment of nuclear transmembrane proteins. MBoC 31:1551–1560. doi:10.1091/mbc.E20-01-0009 PMC7521799 32459568

[39] Li Y, Huang L, Li H, Zhu Y, Yu Z, Zheng X, Weng C, Feng W. 2024. ASFV pA151R negatively regulates type I IFN production via degrading E3 ligase TRAF6. Front Immunol 15:1339510. doi:10.3389/fimmu.2024.1339510 PMC10914938 38449860

[40] Freitas FB, Frouco G, Martins C, Ferreira F. 2019. The QP509L and Q706L superfamily II RNA helicases of African swine fever virus are required for viral replication, having non-redundant activities. Emerg Microbes Infect 8:291–302. doi:10.1080/22221751.2019.1578624 PMC6455146 30866783

[41] Nunes-Correia I, Rodríguez JM, Eulálio A, Carvalho AL, Citovsky V, Simões S, Faro C, Salas ML, Pedroso de Lima MC. 2008. African swine fever virus p10 protein exhibits nuclear import capacity and accumulates in the nucleus during viral infection. Vet Microbiol 130:47–59. doi:10.1016/j.vetmic.2007.12.010 18243588

[42] Eulálio A, Nunes-Correia I, Salas J, Salas ML, Simões S, Pedroso de Lima MC. 2007. African swine fever virus p37 structural protein is localized in nuclear foci containing the viral DNA at early post-infection times. Virus Res 130:18–27. doi:10.1016/j.virusres.2007.05.009 17580096

[43] Eulálio A, Nunes-Correia I, Carvalho AL, Faro C, Citovsky V, Simões S, Pedroso de Lima MC. 2004. Two African swine fever virus proteins derived from a common precursor exhibit different nucleocytoplasmic transport activities. J Virol 78:9731–9739. doi:10.1128/JVI.78.18.9731-9739.2004 PMC514976 15331706

[44] Yang S, Miao C, Liu W, Zhang G, Shao J, Chang H. 2023. Structure and function of African swine fever virus proteins: current understanding. Front Microbiol 14:1043129. doi:10.3389/fmicb.2023.1043129 PMC9950752 36846791

[45] Oh HS, Traktman P, Knipe DM. 2015. Barrier-to-autointegration factor 1 (BAF/BANF1) promotes association of the SETD1A histone methyltransferase with herpes simplex virus immediate-early gene promoters. mBio 6:e00345-15. doi:10.1128/mBio.00345-15 PMC4447252 26015494

[46] Olson AT, Wang Z, Rico AB, Wiebe MS. 2019. A poxvirus pseudokinase represses viral DNA replication via a pathway antagonized by its paralog kinase. PLoS Pathog 15:e1007608. doi:10.1371/journal.ppat.1007608 PMC6395007 30768651

